# Instantaneous death risk, conditional survival and optimal surgery timing in cervical fracture patients with ankylosing spondylitis: A national multicentre retrospective study

**DOI:** 10.3389/fimmu.2022.971947

**Published:** 2022-09-15

**Authors:** Jinfeng Huang, Hao Bai, Quanchang Tan, Dingjun Hao, Aimin Wu, Qingde Wang, Bing Wang, Linfeng Wang, Hao Liu, Xiongsheng Chen, Zhengsong Jiang, Xiaoming Ma, Xinyu Liu, Peng Liu, Weihua Cai, Ming Lu, Ningfang Mao, Yong Wang, Suochao Fu, Shuai Zhao, Xiaofang Zang, Youzhuan Xie, Haiyang Yu, Ruixian Song, Jiangbo Sun, Liangbi Xiang, Xiang Liu, Songkai Li, Bo Liao, Zixiang Wu

**Affiliations:** ^1^ Department of Orthopaedics, Xijing Hospital, The Air Force Medical University, Xi’an, China; ^2^ Department of Spine Surgery, Honghui Hospital, Xi’an Jiaotong University, Xi’an, China; ^3^ Department of Orthopaedics, The Second Affiliated Hospital and Yuying Children’s Hospital of Wenzhou Medical University, Wenzhou, China; ^4^ Department of Spine Surgery, Zhengzhou Orthopaedic Hospital, Zhengzhou, China; ^5^ Department of Orthopaedics, The Second Xiangya Hospital of Central South University, Changsha, China; ^6^ Department of Orthopedics, The Key Laboratory of Orthopedic Biomechanics of Hebei Province, The Third Hospital of Hebei Medical University, Shijiazhuang, China; ^7^ Department of Orthopedic Surgery, West China Hospital, Sichuan University, Sichuan, China; ^8^ Spine Center, Department of Orthopedics, Changzheng Hospital, Second Military Medical University, Shanghai, China; ^9^ Department of Spine Surgery, Shandong Provincial Hospital Affiliated to Shandong First Medical University, Jinan, China; ^10^ Department of Orthopaedics, General Hospital of Ningxia Medical University, Ningxia, China; ^11^ Department of Orthopedic Surgery, Qilu Hospital of Shandong University, Jinan, China; ^12^ Department of Orthopedics, Daping Hospital, Army Medical University, Chongqing, China; ^13^ Department of Orthopaedics, The First Affiliated Hospital of Nanjing Medical University, Nanjing, China; ^14^ Department of Orthopaedics, The First Affiliated Hospital of Anhui Medical University, Hefei, China; ^15^ Department of Spinal Surgery, Changhai Hospital, Second Military Medical University, Shanghai, China; ^16^ Department of Orthopaedics, The First Affiliated Hospital, Sun Yat-sen University, Guangzhou, China; ^17^ Department of Orthopedics, General Hospital of Southern Theater Command of Chinese PLA, Guangzhou, China; ^18^ Department of Orthopaedics, Guangdong Province Hospital of Traditional Chinese Medicine, Guangzhou, China; ^19^ Department of Orthopaedics, The Third Xiangya Hospital of Central South University, Changsha, China; ^20^ Shanghai Key Laboratory of Orthopaedic Implants, Department of Orthopaedic Surgery, Shanghai Ninth People’s Hospital, Shanghai Jiaotong University School of Medicine, Shanghai, China; ^21^ Department of Orthopaedic Surgery, Fuyang People’s Hospital, Fuyang Clinical College of Anhui Medical University, Fuyang, China; ^22^ Department of Orthopedics, PLA 960th Hospital, Jinan City, China; ^23^ Department of Orthopaedics, Shaoyang Zhenggu Hospital, Shaoyang, China; ^24^ Department of Orthopaedics, The General Hospital of Northern Theater Command, Shenyang, China; ^25^ Department of Orthopaedics, Hebei Aidebao Hospital, Langfang, China; ^26^ Department of Spine Surgery, The 940th Hospital of Joint Logistics Support Force of Chinese PLA, Lanzhou, China; ^27^ Department of Orthopaedics Tangdu Hospital, The Air Force Medical University, Xi’an, China

**Keywords:** ankylosing spondylitis, cervical fracture, hazard function, conditional survival, surgery timing

## Abstract

**Background:**

The mortality rate in patients with ankylosing spondylitis (AS) and cervical fracture is relatively high.

**Objectives:**

This study aimed to investigate the instantaneous death risk and conditional survival (CS) in patients with AS and cervical fracture. We also studied the relationship between surgical timing and the incidence of complications.

**Methods:**

This national multicentre retrospective study included 459 patients with AS and cervical fractures between 2003 and 2019. The hazard function was used to determine the risk of instantaneous death. The five-year CS was calculated to show the dynamic changes in prognosis.

**Results:**

The instantaneous death risk was relatively high in the first 6 months and gradually decreased over time in patients with AS and cervical fracture. For patients who did not undergo surgery, the instantaneous risk of death was relatively high in the first 15 months and gradually decreased over time. For patients with American Spinal Injury Association impairment scale (ASIA) A and B, the 5-year CS was 55.3% at baseline, and improved steadily to 88.4% at 2 years. Odds ratios (ORs) for pneumonia, electrolyte disturbance, respiratory insufficiency, and phlebothrombosis decreased as the surgery timing increased.

**Conclusion:**

Deaths occurred mainly in the first 6 months after injury and gradually decreased over time. Our study highlights the need for continued surveillance and care in patients with AS with cervical fractures and provides useful survival estimates for both surgeons and patients. We also observed that early surgery can significantly increase functional recovery, and decrease the incidence of complications and rehospitalisation.

## Introduction

Ankylosing spondylitis (AS) is a chronic inflammatory disorder that primarily affects the spine (1). The global overall incidence of AS ranges from 9 to 30 per 10000; however, its prevalence varies widely in different countries (2). The prevalence of AS in men is much higher than that in women (the male to female ratio is approximately 2–3:1) (3). Chronic back pain and stiffness are the most common symptoms, and any part of the spine may be involved. The biomechanical properties of the spine are altered by chronic inflammation. This chronic process gradually results in spontaneous ossification and fusion of the spinal segments (4). Therefore, this change in the spine can lead to increased susceptibility to vertebral fractures, and even minor injuries can be substantial (4–6).

Patients with AS are nearly 3.5 times more likely to sustain cervical fractures compared to the general population (7, 8). A previous study pointed out that the incidence of vertebral fractures in patients with AS was approximately 10% (9). Among them, nearly 81% of fractures are located at the cervical level (10). Due to the rigid spine in patients with AS, vertebral fractures are often unstable, which causes a high rate of neurological injury, mortality, and morbidity (11, 12). Furthermore, a delay in the diagnosis and timing of surgical intervention can also lead to poor outcomes. Previous studies found a mortality rate of 5.3–11.3% in AS patients with spinal fracture (6, 13). Moreover, the mortality in patients with AS with cervical fracture is nearly double that in patients without AS (14).

For patients with neurological injury, approximately one-third of these patients will experience disease progression without surgery (15). Spinal fractures in patients with AS can lead to devastating complications, and surgery may be the most beneficial approach to prevent these complications (16). It is well known that early surgery can help improve functional recovery; however, whether early surgery could help decrease the incidence of other complications remains unclear.

The instantaneous death risk can reveal the estimated death hazard rates and further the current understanding of how the instantaneous risk of death changed across survival times. Traditional survival analyses mainly estimate the survival rate from the time of diagnosis, such as the 5-year survival rate. However, the mortality risk is the highest during the first few months after injury and gradually decreases over time. Therefore, cumulative survival rate cannot reflect changes in prognosis over time. The concept of conditional survival (CS) can solve this problem by considering only patients who have survived for a certain period, and it may be an appropriate tool to assess dynamic changes in prognosis (17, 18). However, this concept is usually used in cancer research, and we creatively introduce it to assess mortality in cervical fracture patients with AS.

Although, a recent study reported the mortality of cervical fracture patients with AS and diffuse idiopathic skeletal hyperostosis (DISH) (19), information on the characteristics of mortality in patients with cervical fracture and AS is still scarce. Additionally, the instantaneous death risk and CS in patients with cervical fracture and AS have not been studied. Knowing the mortality pattern of cervical fracture patients with AS can further our understanding of the exact survival rate among medium- and long-term survivors, and could promote personalised medicine. Therefore, in the present study, we aimed to show the instantaneous death risk and CS of cervical fracture patients with AS, and to show the importance of early treatment in the prognosis of cervical fracture patients with AS.

## Methods

The study and informed consent were approved by the ethics committee of the Xijing Hospital of Air Force Medical University (KY20212199-F-1).

### Data sources

Data were retrospectively obtained from tertiary care centres, such as Xijing Hospital, West China Hospital, The Third Hospital of Hebei Medical University, Xi’an Honghui Hospital, The First Affiliated Hospital of Sun Yat-sen University, Changhai Hospital of Shanghai, Qilu Hospital of Shandong University, Jiangsu Provincial Hospital, Wenzhou Medical University Second Affiliated Hospital, and Anhui Provincial Hospital. Data were acquired all over the country to ensure that the results were more representative. Written or oral informed patient consent was obtained at the time of admission or at follow-up, when possible.

### Inclusion and exclusion criteria

Patients who admitted to hospitals with AS and cervical fracture between 1 August 2003 and 31 December 2019 were included. AS was diagnosed according to the diagnostic criteria for ankylosing spondylitis (20) and cervical fractures were confirmed by imaging tests (computed tomography (CT) or magnetic resonance imaging (MRI)). For patients with trauma and new pain, imaging tests were performed for fracture screening. Patients with serious infection, tumour, or congenital spinal deformity; patients with blood system diseases (such as hemophilia, primary thrombocytopenic purpura, leukemia, lymphoma, myeloma and aplastic anemia), serious cardiopulmonary disease (such as serious chronic heart failure, acute myocardial infarction, unstable or severe angina pectoris, acute pulmonary embolism, severe chronic obstructive pulmonary disease (COPD) and chronic pulmonary heart disease), or other diseases that may significantly decrease their lifespan; and patients with other spondylitis, such as forestier´s disease or DISH, psoriatic arthritis, inflammatory bowel disease-associated arthritis, and suppurative spondylitis were excluded. Patients who died before hospital admission were excluded from this study.

### Study participants

Ultimately, we included 459 patients who met the inclusion criteria. We collected data, including demographic characteristics, injury mechanism, fracture sites, American Spinal Injury Association impairment scale (ASIA) grade, comorbidities, surgery type, timing, and complications. The study population was followed-up from the date of admission to death or until 31 June 2020 whichever came first. Mortality data were collected by mail, phone calls, short messages, or household registration agencies in government departments.

The choice of surgical procedure, surgical process, postoperative rehabilitation treatment, and orthopaedic treatment are summarised in **Supplement File 1**.

### Variables

At the time of admission, we performed neurological examinations according to the ASIA classification to divide the patients into five subgroups (21). The fracture level was divided into two subgroups based on the fracture sites: the upper cervical spine (C1-C4) and lower cervical spine (C5-C7). Multiple traumas were defined as brain, chest, or other site fractures. Injury patterns were categorised as high-energy injury (e.g. violence, motor vehicle accidents, or falls) and low-energy injury (e.g. fall from standing height or less, recreational activities). All surgeries were performed by experienced spine surgeons.

### Statistical analysis

Survival rates were calculated using the Kaplan–Meier method, and the log-rank test was used to analyse significant differences between the subgroups. We used univariate and multivariate Cox proportional hazard models to determine the risk of mortality. The hazard function for death was estimated using a fixed-bandwidth kernel approach that incorporates boundary kernels (22, 23).

Five-year CS was defined as the probability of survival at five years from the day of diagnosis, given that the patients had already survived for a period of time (17). Therefore, the CS for another Y year was calculated by dividing the survival at (X + Y) years by the survival at X years:


$$CS(Y|X)=\frac{\text{Overall survival}(\text{X}+\text{Y})}{\text{Overall survival}(\text{X})}$$

We calculated the 5-year overall survival at baseline and 5-year CS at 1, 3, 6, and 12 months and 2 years after admission. In addition, CS estimates were stratified according to surgery, multiple severe traumas, and respiratory insufficiency.

Restricted cubic splines (RCS) were used to model the probability of complications according to the time elapsed from injury to surgery. We also examined the non-linear associations between surgical delay and the risk of complications nonparametrically using restricted cubic spline analyses (24). In the cubic spline analysis, we used the earliest surgery timing as the reference and four knots. The RCS was built using STAT software (version 14.2; Stata Corp, College Station, TX), according to a previous guide. RCS is a powerful tool for demonstrating non-linear relationships in regression models (24). In brief, they can show the association between continuous variables and the risks of outcomes.

Statistical analyses were conducted using STAT software (version 14.2; Stata Corp, College Station, TX), SPSS Version 22.0 (IBM Corporation, Armonk, NY), and GraphPad Prism 7.0 (GraphPad Software, San Diego, CA). All *P* values of<0.05 were considered statistically significant in the present study.

## Results

The demographic characteristics of patients are presented in **Table 1**. The mean age (SD) was 52.96 ± 11.65 years, and approximately 94% of the participants were male. The prevalence of ASIA grades A, B, C, D, and E were 19.8%, 38.6%, 15.7%, 8.1%, and 17.9%, respectively. The frequency of surgical treatment among patients with ASIA grades A, B, C, D, and E were 74.7%, 80.2%, 80.6%, 78.4%, and 72.0%, respectively (P=0.556). Hypertension was the most common comorbidity, whereas pneumonia and respiratory disease were the most common complications. Furthermore, nearly 77.6% of patients underwent surgical treatment. Among them, 22.9% of patients underwent anterior surgical treatment, 34.4% were treated with posterior surgery, and 20.3% were treated with combined anterior-posterior surgery.

**Table 1:** Baseline characteristics of study participants (n=459).

	No. (%)
**Demographics**	
**Age, year**	
Mean (SD)	52.96 (11.65)
Median (IQR)	52.0 (45.0-59.0)
**Sex**	
Male	433 (94.3%)
Female	26 (5.7%)
**Injury factors**	
High-energy injury	136 (29.6%)
Low-energy injury	323 (70.4%)
**Fracture level**	
Upper cervical spine (C1-C4)	142 (30.9%)
Lower cervical spine (C5-C7)	317 (69.1%)
**ASIA grade**	
A	91 (19.8%)
B	177 (38.6%)
C	72 (15.7%)
D	37( 8.1%)
E	82 (17.9%)
**Associated conditions**	
Brain injury	20 (4.4%)
Chest injury	29 (6.3%)
Fractures in other parts	70 (15.3%)
**Comorbidities**	
0	379 (82.6%)
1	55 (12.0%)
2	6 (1.3%)
≥3	19 (4.1%)
**Surgery type**	
Anterior	105 (22.9%)
Posterior	158 (34.4%)
Combined	93 (20.3%)
Non-surgery	103 (22.4%)
**Time-to-operation, h**	
Mean (SD)	29.13 (64.09)
Median (IQR)	15 (8-30)
**Hospitalization Characteristics**	
Length of stay, day (SD)	17.64 (16.90)
Rehospitalization	269 (58.6%)
**Complications**	
Pneumonia	95 (20.7%)
Pleural effusion	30 (6.5%)
Respiratory failure	62 (13.5%)
Deep venous thrombosis	16 (3.5%)
Electrolyte disturbance	32 (7.0%)
Loose internal fixation/Fracture displacement	10 (2.2%)
Decubitus	12 (2.6%)
Urinary tract infection	10 (2.2%)
Digestive system disorder	14 (3.1%)
Wound infection	6 (1.3%)

ASIA, American Spinal Injury Association impairment scale; SD, standard deviation; IQR, interquartile range.

**Figure 1:**
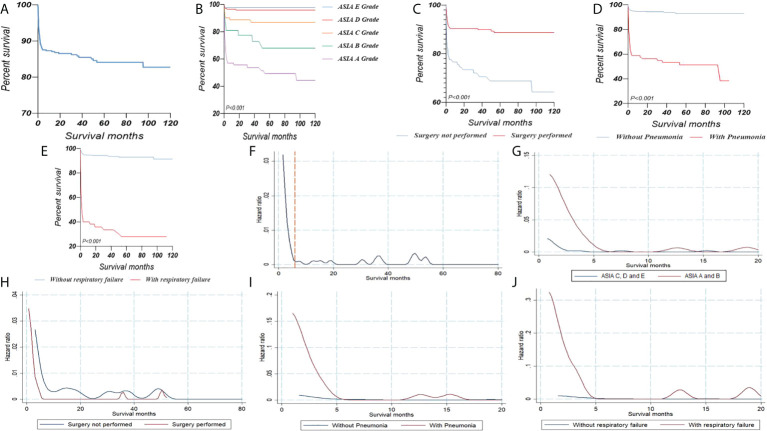
**(A)** Kaplan–Meier survival curves illustrating the overall survival of cervical fracture patients with AS. Kaplan–Meier survival curves stratified by ASIA grade **(B)**, surgery **(C)**, pneumonia **(D)** and respiratory failure **(E)**. Hazard functions for death in cervical fracture patients with AS **(F)**. Hazard functions curve stratified by ASIA grade **(G)**, surgery **(H)**, pneumonia **(I)** and respiratory failure **(J)**. AS, ankylosing spondylitis; ASIA, American Spinal Injury Association impairment scale.

The overall Kaplan–Meier survival curve is shown in **Figure 1A**. When classified by ASIA grade, we found deaths mainly in patients with ASIA grades A and B (**Figure 1B**). In addition, we found that patients with pneumonia or respiratory failure had a significantly poor prognosis, whereas surgery could significantly improve prognosis (**Figures 1C–E**). We then used the hazard function curve to show the hazard rates over time, and the instantaneous death risk among the survivors changed over time (**Figures 1F–J**). For the total population, we found that the instantaneous death risk was relatively high in the first 6 months and gradually decreased over time. Similarly, we found that the instantaneous death risk among patients with ASIA A and B, pneumonia, and respiratory failure was much higher. However, surgery can help decrease the risk of instantaneous death more rapidly.

The results of the multivariate Cox regression analysis are presented in **Table 2**. For the total population, we found that age, ASIA A and B grade, non-surgery, pneumonia, pleural effusion, respiratory insufficiency, deep venous thrombosis, electrolyte disturbance, decubitus, urinary tract infection, and digestive system disorders were risk factors (P<0.05). However, for patients who had already survived for 1 month, we found that only age, non-surgery, pneumonia, respiratory insufficiency, and digestive system disorder were risk factors (P<0.05). In addition, for patients who had already survived for 3 months, we found that only surgery, pneumonia, respiratory insufficiency, and deep venous thrombosis significantly influenced prognosis (P<0.05).

**Table 2:** COX regression results of the risk factors of mortality in baseline, ≥ 1- and 3-month survivors.

	Baseline				≥ 1-Month Survivors				≥ 3-Month Survivors			
	Univariable COX regression		Multivariable COX regression		Univariable COX regression		Multivariable COX regression		Univariable COX regression		Multivariable COX regression	
Covariables	Hazard Ratio (95% CI)	P-value	Hazard Ratio (95% CI)	P-value	Hazard Ratio (95% CI)	P-value	Hazard Ratio (95% CI)	P-value	Hazard Ratio (95% CI)	P-value	Hazard Ratio (95% CI)	P-value
**Age**	1.042 (1.021-1.063)	<0.001	1.031 (1.008-1.054)	0.008	1.038 (1.010-1.067)	0.008	1.045 (1.010-1.082)	0.024	1.029 (0.987-1.074)	0.178	–	–
**Gender**												
Male	REF	REF	–	–	–	–	–	–	–	–	–	–
Female	0.251 (0.035-1.811)	0.171	–	–	–	–	–	–	–	–	–	–
**Fracture level**												
Upper cervical spine (C1-C4)	REF	REF	–	–	REF	REF	REF	REF	REF	REF	–	–
Lower cervical spine (C5-C7)	1.755 (0.975-3.158)	0.061	–	–	2.982 (1.162-7.653)	0.023	1.987 (0.712-5.543)	0.190	3.344 (0.760-14.715)	0.110	–	–
**Injury factors**												
Low-energy injury	REF	REF	–	–	REF	REF	–	–	REF	REF	–	–
High-energy injury	1.012 (0.654-1.568)	0.956	–	–	1.175 (0.651-2.121)	0.592	–	–	1.450 (0.594-3.541)	0.415	–	–
**ASIA grade**												
E	REF	REF	REF	REF	REF	REF	REF	REF	–	–	–	–
D	1.807 (0.375-8.697)	0.461	1.588 (0.326-7.731)	0.567	2.063 (0.231-18.460)	0.517	1.583 (0.172-14.579)	0.685	–	–	–	–
C	5.731 (1.238-26.528)	0.026	3.089 (0.636-15.011)	0.162	5.054 (0.565-45.236)	0.147	2.186 (0.227-21.022)	0.498	–	–	–	–
B	13.504 (2.959-61.635)	0.001	6.036 (1.260-28.929)	0.025	19.561 (2.407-159.003)	0.005	7.226 (0.829-62.991)	0.073	–	–	–	–
A	28.899 (6.981-119.638)	<0.001	6.606 (1.446-30.187)	0.015	34.357 (4.620-255.484)	0.001	5.303 (0.617-45.554)	0.128	–	–	–	–
**Comorbidities**												
0	REF	REF	REF	REF	REF	REF	REF	REF	–	–	–	–
1	0.788 (0.339-1.833)	0.580	0.672 (0.238-1.896)	0.452	0.530 (0.074-3.805)	0.159	0.209 (0.024-1.848)	0.159	–	–	–	–
2	2.547 (0.620-10.463)	0.195	0.327 (0.077-1.388)	0.130	5.103 (1.214-21.458)	0.026	0.548 (0.120-2.502)	0.437	–	–	–	–
≥3	2.403 (1.034-5.588)	0.042	0.996 (0.381-2.607)	0.994	2.991 (1.053-8.495)	0.040	0.879 (0.233-3.314)	0.849	–	–	–	–
**Surgery**												
Surgery not performed	REF	REF	REF	REF	REF	REF	REF	REF	REF	REF	REF	REF
Surgery performed	0.315 (0.195-0.507)	<0.001	0.293 (0.171-0.504)	<0.001	0.338 (0.177-0.649)	0.001	0.287 (0.129-0.637)	0.002	0.157 (0.057-0.431)	<0.001	0.140 (0.045-0.433)	0.001
**Complications**												
Pneumonia	9.719 (5.865-16.105)	<0.001	2.491 (1.326-4.681)	0.005	10.551 (5.357-20.780)	<0.001	3.130 (1.361-7.197)	0.007	8.249 (3.055-22.275)	<0.001	3.137 (1.022-9.624)	0.046
Pleural effusion	2.319 (1.149-4.680)	0.019	0.939 (0.428-2.061)	0.876	4.483 (2.044-9.830)	<0.001	1.322 (0.497-3.516)	0.576	2.712 (0.614-11.976)	0.188	–	–
Respiratory failure	17.072 (10.398-28.029)	<0.001	5.146 (2.770-9.560)	<0.001	14.323 (7.488-27.396)	<0.001	4.626 (2.037-10.507)	<0.001	12.921 (4.798-34.798)	<0.001	5.611 (1.873-16.804)	0.002
Deep venous thrombosis	3.779 (1.726-8.272)	0.001	0.780 (0.306-1.979)	0.602	4.440 (1.570-12.562)	0.005	0.717 (0.205-2.514)	0.603	10.176 (2.879-35.971)	<0.001	4.520 (1.180-17.320)	0.028
Electrolyte disturbance	4.201 (2.322-7.600)	<0.001	0.980 (0.485-1.979)	0.954	7.366 (3.522-15.406)	<0.001	1.415 (0.592-3.379)	0.435	6.981 (1.951-24.978)	0.003	3.091 (0.650-14.688)	0.156
Decubitus	4.589 (1.983-10.619)	<0.001	2.611 (1.050-6.492)	0.039	4.602 (1.412-15.003)	0.011	2.754 (0.735-10.314)	0.133	–	–	–	–
Urinary tract infection	3.217 (1.171-8.837)	0.023	0.424 (0.120-1.494)	0.182	3.290 (0.791-13.680)	0.101	–	–	3.989 (0.527-30.208)	0.180	–	–
Digestive system disorder	6.062 (2.891-12.715)	<0.001	4.504 (1.900-10.678)	0.001	4.932 (1.511-16.099)	0.008	12.363 (2.952-51.776)	0.001	–	–	–	–

ASIA, American Spinal Injury Association impairment scale; CI, confidence index; REF, reference.

Because mortality was relatively high in patients with ASIA grades A and B, we calculated the CS for this population. The 5-year CS increased from 55.3% at baseline to 88.4% at 2 years (**Table 3** and **Supplement Figure 1**). The 5-year CS was much lower in non-surgical patients but improved a lot after surviving for two years. (**Supplement Figure 1B**). Pneumonia had a significant influence on prognosis at baseline. After survival for 6 months, the 5-year CSs for these two groups were higher than 80% (**Supplement Figure 1C**). Additionally, we found that respiratory insufficiency significantly increased patient mortality. Subjects who had respiratory insufficiency had the lowest relative survival at baseline (25.5%), but the survival rate increased rapidly over time. After surviving for 2 years, the 5-year CS rate for patients with respiratory insufficiency was > 70%.

**Table 3:** Five-year conditional survival rates among ASIA A and B grade patients.

		Conditional 5-year relative survival (%)				
Variables	5-Year relative survival at baseline (%)	At 1 month	At 3 months	At 6 months	At 1 year	At 2 years
**Total**	55.3 ± 5.3	67.8 ± 5.4	81.3 ± 5.3	85.6 ± 5.1	85.6 ± 5.1	88.4 ± 4.9
**Surgery performed**						
Yes	63.6 ± 5.6	74.7 ± 5.6	88.9 ± 5.0	93.4 ± 4.5	93.4 ± 4.5	93.4 ± 4.5
No	35.2 ± 9.2	49.6 ± 11.7	60.6 ± 12.9	64.2 ± 13.1	64.2 ± 13.1	72.7 ± 13.4
**Pneumonia**						
Yes	42.4 ± 6.8	55.3 ± 8.0	77.1 ± 8.5	85.1 ± 8.0	85.1 ± 8.0	88.8 ± 7.5
No	68.1 ± 7.0	78.8 ± 7.1	83.7 ± 6.9	85.5 ± 6.8	85.5 ± 6.8	87.8 ± 6.6
**Respiratory insufficiency**						
Yes	25.5 ± 6.8	38.2 ± 9.4	58.4 ± 12.2	64.9 ± 12.7	64.9 ± 12.7	75.0 ± 12.5
No	76.5 ± 5.6	83.5 ± 5.3	90.5 ± 4.7	93.6 ± 4.4	93.6 ± 4.4	93.6 ± 4.4

ASIA, American Spinal Injury Association impairment scale.

**Figure 2:**
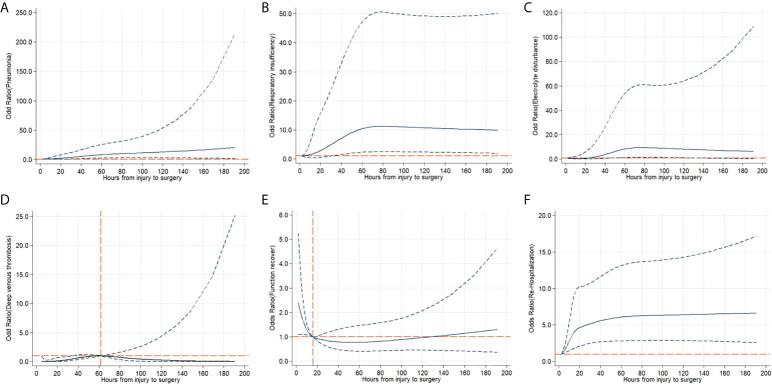
Adjusted cubic spline models showing the association between surgery timing and the incidence of complications **(A-D)**, functional recovery **(E)** and rehospitalization **(F)**. Models were adjusted for age, sex, injury factors, fracture level and comorbidities. The solid line and long dashed line represent the estimated odds ratio and its 95% confidence interval. Knots are at the 5th, 35th, 65th and 95th percentiles for metabolic equivalents.

The relationships between surgical timing and complications, functional recovery, and rehospitalisation were analysed using restricted cubic spline models. The odds ratios (ORs) for pneumonia, electrolyte disturbance, respiratory insufficiency, and phlebothrombosis decreased as surgery timing increased. This suggests that early surgery may decrease the prevalence of complications (**Figures 2A–D**). In addition, early surgery increased the probability of functional recovery (**Figure 2E**). We also found that early surgery decreased the incidence of re-hospitalization (**Figure 2F**).

## Discussion

To the best of our knowledge, this is the first study to assess the instantaneous death risk and CS among patients with AS and cervical fracture. In addition, we investigated the relationship between surgical timing and complications, functional recovery, and rehospitalisation. We found that the instantaneous death risk was relatively high in the first 6 months after injury and gradually decreased over time. For patients who did not undergo surgery, the instantaneous risk of death was relatively high in the first 15 months and gradually decreased over time. The 5-year CS of AS patients with cervical fractures has steadily improved. For patients who survived for more than 6 months, the 5-year survival rate increased by nearly 30% compared with the baseline survival rate.

The innovations and advantages of our study can be summarised as follows: 1) Our study had a relatively large sample size and a long follow-up time; 2) our study showed that the instantaneous death risk patients faced in different time periods has not been studied previously; 3) our study provided a conditional survival rate which helps us understand the exact survival rate among medium- and long-term survivors; and 4) our results do help us know dynamic changes in prognosis and meanwhile promote personalized medicine.

A previous study indicated that cervical fractures are most common in patients with AS (6). In addition, they are often unstable fractures with a high risk of spinal cord injury (SCI). Patients with SCI have a poorer prognosis and a higher risk of mortality and morbidity (25). This result was similar to our findings that deaths occurred were mainly in patients with ASIA grades A and B. Previous studies pointed out that the in-hospital mortality rate was approximately 3.4–17% (14, 25–29). However, the above studies may be underpowered because they only assessed in-hospital mortality with a very short time period and had a relatively low number of patients. Therefore, further studies are warranted to describe mortality in AS patients with cervical fracture (30). Our study provided detailed mortality data for patients with AS and cervical fracture. The follow-up period was relatively long and the number of participants was relatively large.

A previous study indicated that the prognosis of patients with SCI is associated with the neurological level of injury (NLI) (31). Besides, Groah et al. reported that NLI contributed to the risk of cardiovascular disease in patients with SCI (32). A more recent study showed that older patients with injuries at levels higher than C4 were nearly seven times more likely to die than those with cervical SCI lower than C4 (33). Generally, NLI and different fracture type may be closely associated with mortality and complications. According to the AO classification system, cervical fractures among patients with AS are commonly type B or C fractures which are usually unstable. Classification of cervical fractures can help in treatment choice (34). However, information regarding the prognosis of different AO cervical fracture types remains limited. Therefore, in future studies, we would like to clarify the association between NLI and different AO fracture types and prognoses among patients with AS.

Although the survival function is fundamental in conveying how mortality risk changes over time, the hazard function provides additional useful insights over and above what can be easily obtained from the survival function (35). It can be used to obtain more detailed information regarding the insufficiency process for patients. The hazard function conveys how risk changes over time in terms of instantaneous death risk among survivors (36). Therefore, the hazard function can provide significant clinical insights. To the best of our knowledge, this study is the first to provide a hazard function for patients with AS and cervical fractures. From our results, we can easily determine the change in the instantaneous death risk over time and the period with the highest death risk.

Baseline survival prediction may play a significant role in determining treatment options and life planning. However, its prognostic accuracy may be lost when patients outlive their initially predicted survival time (37). Our results demonstrated changes in the probability of survival over time after initial survival projections. This statistical concept provides dynamic prognosis prediction (38). We found that the increase in CS was the highest among patients with respiratory insufficiency, with the least favourable prognosis at baseline. In addition, CS significantly increased in patients with pneumonia and in those who could not receive surgical treatment. For these groups, who survived for more than 6 months, we can confidently state that the five-year survival rate is nearly 80% instead of 30–40%. Our results will certainly assure patients of their likelihood to live longer.

A recent study revealed that patients with AS with cervical fracture, especially ASIA A, are at a high risk of complications (29). There is growing concern about the complications in AS patients receiving surgical treatment for spine fractures. However, there is limited research in the literature. Previous studies have pointed out that surgical site infection, implant insufficiency, and nosocomial pneumonia were the main complications during hospitalisation (27, 29, 39–43). In contrast to earlier findings, we found that pulmonary complications were the most common complications and significantly affected prognosis. One possible explanation is that our study had a long follow-up period. In addition, several studies grouped AS and DISH as ankylosing spinal disorders for further research, which may limit the ability to obtain AS-specific results. Therefore, our results provide an avenue for the management of complications in patients receiving surgical treatment for spinal fractures. In addition, the relationship between surgery timing and the incidence of complications has not been studied. We first pointed out that early surgery can significantly increase functional recovery and decrease the incidence of complications and rehospitalisation. Finally, only surgical treatment remained an independent predictor of survival at baseline and at one- and 3-month survivorships. These results show the importance of early surgery. Therefore, our results are important for providing prognostic information not only for patients but also for surgeons.

Previous studies have shown the rate of delay in diagnosing fractures in patients with a rigidspine was very high (39, 44). Over 80% of patients have a secondary decline in neurologic function when there is a delay in diagnosis (39). Therefore, early diagnosis and intervention in AS patients with cervical fractures may minimise the risk of complications (45). Similarly, Our results partially support this conclusion. Early diagnosis and treatment are very important for patients with AS and cervical fractures.

However, this study has some limitations. First, the inherent limitations of a retrospective study may have led to an underestimation of mortality and comorbidities. Second, because the CS model cannot incorporate multivariate analysis, the estimates may have been be slightly biased. This limitation can be partially overcome by conducting stratified analyses to minimise the effects of relevant characteristics. Third, the survival rate was very high in patients with ASIA grades E and D. Therefore, we could not include these patients in further survival analysis. Fourth, we inevitably missed deaths before admission, which may have overestimated the survival rate. Fifth, every country has a different healthcare system; therefore, our data may not be easily generalisable to other countries. Finally, there are still some differences in the cognition and judgment of different surgeons between different hospitals, which may affect patients’ prognosis.

## Conclusion

We found that in patients with AS with cervical fractures, the instantaneous death risk was relatively high in the first 6 months and gradually decreased over time. For patients who did not undergo surgery, the instantaneous risk of death was relatively high in the first 15 months and gradually decreased over time. Additionally, the 5-year CS of AS patients with cervical fractures has improved over time. The largest improvements in CS were observed in the patients with respiratory insufficiency. Our study highlights the need for continued surveillance and care in patients with AS with cervical fractures and provides useful survival estimates for both surgeons and patients. We also indicated that early surgery can significantly increase functional recovery and decrease the incidence of complications and rehospitalisation. Therefore, early diagnosis and treatment (surgical or orthopaedic), and an intense rehabilitation protocol may be beneficial for these patients.

## Data availability statement

The raw data supporting the conclusions of this article will be made available by the authors, without undue reservation.

## Ethics statement

The studies involving human participants were reviewed and approved by Xijing Hospital of Air Force Medical University. The patients/participants provided their oral/written informed consent to participate in this study.

## Author contributions

JH, HB, QT, BL and ZW had the original idea for the study, contributed to the development of the study, constructed the study design and the statistical model, reviewed the literature, and act as guarantors for the study. JH, HB and QT wrote the first draft of the manuscript. ZW is the principal investigator and provided oversight for all aspects of this project. All authors provided critical input to the analyses, design and discussion. All authors contributed to the interpretation of the analysis, critically reviewed and revised the manuscript, and approved the final manuscript as submitted.

## Conflict of interest

The authors declare that the research was conducted in the absence of any commercial or financial relationships that could be construed as a potential conflict of interest.

## Publisher’s note

All claims expressed in this article are solely those of the authors and do not necessarily represent those of their affiliated organizations, or those of the publisher, the editors and the reviewers. Any product that may be evaluated in this article, or claim that may be made by its manufacturer, is not guaranteed or endorsed by the publisher.

## References

[1] Braun J Sieper J . Ankylosing spondylitis. Lancet (2007) 369(9570):1379–90. doi: 10.1016/s0140-6736(07)60635-7 17448825

[2] Wang R Ward MM . Epidemiology of axial spondyloarthritis: an update. Curr Opin Rheumatol (2018) 30(2):137–43. doi: 10.1097/bor.0000000000000475 PMC5811203 29227352

[3] van Tubergen A . The changing clinical picture and epidemiology of spondyloarthritis. Nat Rev Rheumatol (2015) 11(2):110–8. doi: 10.1038/nrrheum.2014.181 25385414

[4] Shah NG Keraliya A Nunez DB Schoenfeld A Harris MB Bono CM . Injuries to the rigid spine: What the spine surgeon wants to know. Radiographics (2019) 39(2):449–66. doi: 10.1148/rg.2019180125 30707647

[5] Jacobs WB Fehlings MG . Ankylosing spondylitis and spinal cord injury: origin, incidence, management, and avoidance. Neurosurg Focus (2008) 24(1):E12. doi: 10.3171/foc/2008/24/1/e12 18290738

[6] Westerveld LA Verlaan JJ Oner FC . Spinal fractures in patients with ankylosing spinal disorders: a systematic review of the literature on treatment, neurological status and complications. Eur Spine J (2009) 18(2):145–56. doi: 10.1007/s00586-008-0764-0 PMC2899332 18791749

[7] Surin VV . Fractures of the cervical spine in patients with ankylosing spondylitis. Acta Orthop Scand (1980) 51(1):79–84. doi: 10.3109/17453678008990772 7376849

[8] Rowed DW . Management of cervical spinal cord injury in ankylosing spondylitis: the intervertebral disc as a cause of cord compression. J Neurosurg (1992) 77(2):241–6. doi: 10.3171/jns.1992.77.2.0241 1625012

[9] Davey-Ranasinghe N Deodhar A . Osteoporosis and vertebral fractures in ankylosing spondylitis. Curr Opin Rheumatol (2013) 25(4):509–16. doi: 10.1097/BOR.0b013e3283620777 23719363

[10] Samartzis D Anderson DG Shen FH . Multiple and simultaneous spine fractures in ankylosing spondylitis: case report. Spine (Phila Pa 1976) (2005) 30(23):E711–715. doi: 10.1097/01.brs.0000188272.19229.74 16319741

[11] Thumbikat P Hariharan RP Ravichandran G McClelland MR Mathew KM . Spinal cord injury in patients with ankylosing spondylitis: a 10-year review. Spine (Phila Pa 1976) (2007) 32(26):2989–95. doi: 10.1097/BRS.0b013e31815cddfc 18091492

[12] Leone A Marino M Dell’Atti C Zecchi V Magarelli N Colosimo C . Spinal fractures in patients with ankylosing spondylitis. Rheumatol Int (2016) 36(10):1335–46. doi: 10.1007/s00296-016-3524-1 27379763

[13] Hoh DJ Rahman M Fargen KM Neal D Hoh BL . Establishing standard hospital performance measures for cervical spinal trauma: a nationwide in-patient sample study. Spinal Cord (2016) 54(4):306–13. doi: 10.1038/sc.2015.185 26481701

[14] Wysham KD Murray SG Hills N Yelin E Gensler LS . Cervical spinal fracture and other diagnoses associated with mortality in hospitalized ankylosing spondylitis patients. Arthritis Care Res (Hoboken) (2017) 69(2):271–7. doi: 10.1002/acr.22934 PMC5102813 27159625

[15] Ramos-Remus C Gomez-Vargas A Hernandez-Chavez A Gamez-Nava JI Gonzalez-Lopez L Russell AS . Two year followup of anterior and vertical atlantoaxial subluxation in ankylosing spondylitis. J Rheumatol (1997) 24(3):507–10. 9058657

[16] El Tecle NE Abode-Iyamah KO Hitchon PW Dahdaleh NS . Management of spinal fractures in patients with ankylosing spondylitis. Clin Neurol Neurosurg (2015) 139:177–82. doi: 10.1016/j.clineuro.2015.10.014 26513429

[17] Skuladottir H Olsen JH . Conditional survival of patients with the four major histologic subgroups of lung cancer in Denmark. J Clin Oncol (2003) 21(16):3035–40. doi: 10.1200/jco.2003.04.521 12915592

[18] Fuller CD Wang SJ Thomas CR Jr. Hoffman HT Weber RS Rosenthal DI . Conditional survival in head and neck squamous cell carcinoma: results from the SEER dataset 1973-1998. Cancer (2007) 109(7):1331–43. doi: 10.1002/cncr.22563 17326199

[19] Romero-Muñoz LM Tipper G Segura-Fragoso A Barriga-Martín A . Outcomes of spinal cord injury following cervical fracture in ankylosing spondylitis and diffuse idiopathic skeletal hyperostosis (DISH): A prospective cohort study. Neurocirugía (2021). doi: 10.1016/j.neucir.2021.06.004 36333086

[20] van der Linden S Valkenburg HA Cats A . Evaluation of diagnostic criteria for ankylosing spondylitis. a proposal for modification of the new York criteria. Arthritis Rheum (1984) 27(4):361–8. doi: 10.1002/art.1780270401 6231933

[21] Middleton JW Dayton A Walsh J Rutkowski SB Leong G Duong S . Life expectancy after spinal cord injury: a 50-year study. Spinal Cord (2012) 50(11):803–11. doi: 10.1038/sc.2012.55 22584284

[22] Müller HG Wang JL . Hazard rate estimation under random censoring with varying kernels and bandwidths. Biometrics (1994) 50(1):61–76. doi: 10.2307/2533197 8086616

[23] Hess KR Serachitopol DM Brown BW . Hazard function estimators: a simulation study. Stat Med (1999) 18(22):3075–88. doi: 10.1002/(sici)1097-0258(19991130)18:22<3075::aid-sim244>3.0.co;2-6 10544307

[24] Harrell FE Jr. Lee KL Pollock BG . Regression models in clinical studies: determining relationships between predictors and response. J Natl Cancer Inst (1988) 80(15):1198–202. doi: 10.1093/jnci/80.15.1198 3047407

[25] Lukasiewicz AM Bohl DD Varthi AG Basques BA Webb ML Samuel AM . Spinal fracture in patients with ankylosing spondylitis: Cohort definition, distribution of injuries, and hospital outcomes. Spine (Phila Pa 1976) (2016) 41(3):191–6. doi: 10.1097/brs.0000000000001190 26579959

[26] Schoenfeld AJ Harris MB McGuire KJ Warholic N Wood KB Bono CM . Mortality in elderly patients with hyperostotic disease of the cervical spine after fracture: an age- and sex-matched study. Spine J (2011) 11(4):257–64. doi: 10.1016/j.spinee.2011.01.018 21377938

[27] Westerveld LA van Bemmel JC Dhert WJ Oner FC Verlaan JJ . Clinical outcome after traumatic spinal fractures in patients with ankylosing spinal disorders compared with control patients. Spine J (2014) 14(5):729–40. doi: 10.1016/j.spinee.2013.06.038 23992936

[28] Dhital R Oke I Donato A Paudel A Poudel DR Paudel P . Trends in hospitalizations for vertebral compression fracture in ankylosing spondylitis: data from the national inpatient sample 2000-2014. Clin Rheumatol (2021) 40(12):4927–32. doi: 10.1007/s10067-021-05842-0 34224028

[29] Ull C Yilmaz E Hoffmann MF Reinke C Aach M Schildhauer TA . Factors associated with major complications and mortality during hospitalization in patients with ankylosing spondylitis undergoing surgical management for a spine fracture. Global Spine J (2022) 12(7):1380–7. doi: 10.1177/2192568220980702 33430630 PMC9394001

[30] Ognjenovic M Raymond WD Inderjeeth CA Keen HI Preen DB Nossent JC . The risk and consequences of vertebral fracture in patients with ankylosing spondylitis: A population-based data linkage study. J Rheumatol (2020) 47(11):1629–36. doi: 10.3899/jrheum.190675 32062601

[31] Wilson JR Jaja BNR Kwon BK Guest JD Harrop JS Aarabi B . Natural history, predictors of outcome, and effects of treatment in thoracic spinal cord injury: A multi-center cohort study from the north American clinical trials network. J Neurotrauma (2018) 35(21):2554–60. doi: 10.1089/neu.2017.5535 29665733

[32] Groah SL Weitzenkamp D Sett P Soni B Savic G . The relationship between neurological level of injury and symptomatic cardiovascular disease risk in the aging spinal injured. Spinal Cord (2001) 39(6):310–7. doi: 10.1038/sj.sc.3101162 11438852

[33] Daneshvar P Roffey DM Brikeet YA Tsai EC Bailey CS Wai EK . Spinal cord injuries related to cervical spine fractures in elderly patients: factors affecting mortality. Spine J (2013) 13(8):862–6. doi: 10.1016/j.spinee.2013.01.045 23453576

[34] Schroeder GD Canseco JA Patel PD Divi SN Karamian BA Kandziora F . Establishing the injury severity of subaxial cervical spine trauma: Validating the hierarchical nature of the AO spine subaxial cervical spine injury classification system. Spine (Phila Pa 1976) (2021) 46(10):649–57. doi: 10.1097/brs.0000000000003873 PMC8057527 33337687

[35] Silcocks P . Hazard ratio funnel plots for survival comparisons. J Epidemiol Community Health (2009) 63(10):856–61. doi: 10.1136/jech.2008.075069 19542076

[36] Hess KR Levin VA . Getting more out of survival data by using the hazard function. Clin Cancer Res (2014) 20(6):1404–9. doi: 10.1158/1078-0432.ccr-13-2125 24501392

[37] Kang M Park JY Jeong CW Hwang EC Song C Hong SH . Changeable conditional survival rates and associated prognosticators in patients with metastatic renal cell carcinoma receiving first line targeted therapy. J Urol (2018) 200(5):989–95. doi: 10.1016/j.juro.2018.06.030 29940249

[38] Zabor EC Gonen M Chapman PB Panageas KS . Dynamic prognostication using conditional survival estimates. Cancer (2013) 119(20):3589–92. doi: 10.1002/cncr.28273 23913639

[39] Caron T Bransford R Nguyen Q Agel J Chapman J Bellabarba C . Spine fractures in patients with ankylosing spinal disorders. Spine (Phila Pa 1976) (2010) 35(11):E458–464. doi: 10.1097/BRS.0b013e3181cc764f 20421858

[40] Schiefer TK Milligan BD Bracken CD Jacob JT Krauss WE Pichelmann MA . In-hospital neurologic deterioration following fractures of the ankylosed spine: a single-institution experience. World Neurosurg (2015) 83(5):775–83. doi: 10.1016/j.wneu.2014.12.041 25545552

[41] Teunissen FR Verbeek BM Cha TD Schwab JH . Spinal cord injury after traumatic spine fracture in patients with ankylosing spinal disorders. J Neurosurg Spine (2017) 27(6):709–16. doi: 10.3171/2017.5.spine1722 28984512

[42] Vazan M Ryang YM Barz M Török E Gempt J Meyer B . Ankylosing spinal disease-diagnosis and treatment of spine fractures. World Neurosurg (2019) 123:e162–70. doi: 10.1016/j.wneu.2018.11.108 30476662

[43] Bernstein DN McCalla DJ Molinari RW Rubery PT Menga EN Mesfin A . An analysis of patient and fracture characteristics and clinical outcomes in patients with hyperostotic spine fractures. Global Spine J (2020) 10(8):964–72. doi: 10.1177/2192568219887157 PMC7645086 32875832

[44] Hartmann S Tschugg A Wipplinger C Thomé C . Analysis of the literature on cervical spine fractures in ankylosing spinal disorders. Global Spine J (2017) 7(5):469–81. doi: 10.1177/2192568217700108 PMC5544161 28811992

[45] Ren C Zhu Q Yuan H . Imaging features of spinal fractures in ankylosing spondylitis and the diagnostic value of different imaging methods. Quant Imaging Med Surg (2021) 11(6):2499–508. doi: 10.21037/qims-20-962 PMC8107346 34079719

