# Supplementary material for: Instantaneous death risk, conditional survival and optimal surgery timing in cervical fracture patients with ankylosing spondylitis: A national multicentre retrospective study
Source: Front Immunol. 2022 Sep 15;13:971947. doi: 10.3389/fimmu.2022.971947 (PMC9521542; doi:10.3389/fimmu.2022.971947)
Supplement: Supplementary file 1 [file DataSheet_1.docx]

***Surgical procedure choice***

Generally, nonsurgical treatment of cervical fractures among AS patients are rarely recommended. Only patients with simple AO spine A-type fractures, with surgical contraindications, or who refused surgery may be treated nonoperatively. Patients who underwent nonsurgical treatment were immobilised in a halo-vest treatment or cervical collar according to their specific condition. Orthotic extension to the thoracic and even lumbar spine may be selected depending on the area of instability. For nonsurgical treatment, close follow-up of all caregivers regarding alignment changes and neurologic decline is needed.

However, cervical fractures in patients were highly unstable. The common reasons for surgical management include neurologic deterioration, presence of an unstable fracture (Most are AO Spine B- and C-type cervical fractures) or detection of an epidural haematoma.

Surgical procedure choice includes an anterior-only procedure, a posterior-only procedure, and combined anterior-posterior surgery. The choice of the surgical procedure is complicated.

For patients with only anterior column injury, intervertebral disc rupture, or without obvious deformity and dislocation, an anterior-only procedure can be considered.

If the anterior axial load-bearing column is competent and without anterior spinal cord compression, a posterior-only procedure can be considered. For posterior-only procedures, long constructs bypassing the fracture ideally encompassing at least three to four segments of rostral and distal fixation are usually recommended.

For patients with fractures involving all three columns, unstable cervical fractures and deformities, or with both anterior and posterior spinal cord compression, and combined anterior-posterior surgery should be conducted.

Most cervical fractures in patients with AS require surgical treatment. Surgical choice should be based on many factors, such as injury type, stability of fracture, spinal cord compression, dislocation, and deformity.

***Postoperative rehabilitation treatment***

After surgical treatment, postoperative cervical immobilisation was achieved using a halo-vest, cervical collar, or sterno-occipital mandibular immobilisation device for at least one month. Common postoperative rehabilitation treatments, such as pulmonary rehabilitation, urinary tract management, skin care, and training of muscle strength, range of joint motion, joint position sense, and gait (if possible) were conducted.

***Surgical Procedure***

For the anterior approach, the right-neck oblique approach is usually used to expose the longitudinal ligament and fracture site of the cervical spine. Next, complete bone graft fusion and anterior cervical plate fixation were performed to fix both the upper and lower sides. For the posterior approach, a posterior median incision was made and the vertebral laminae were clearly exposed. Soft tissue injury and fracture morphology were assessed. After careful estimation, cervical lateral mass screws and upper thoracic pedicle screws (if necessary) were inserted on both sides with a fixation number of at least two above and two below the injured level. If compression or a haematoma exists, surgeons may perform laminectomy according to specific conditions. Finally, the allograft bone was used for posterolateral fusion. For combined anteroposterior surgery, anterior and posterior procedures were performed based on the above process. The sequence of anterior and posterior procedures depended on specific conditions.

***Orthopaedic treatment***

Some patients whose sagittal balance was maintained or restored through surgical treatment but whose kyphotic chin-brow angle has severely impaired the visual field or interfered with either hygiene or swallowing can be treated with a gradual halo-vest to restore accurate alignment of the spinal canal and chin-brow angle. The correction procedure must be adapted to the patient's tolerance and daily monitoring.
