# Supplementary material for: Instantaneous death risk, conditional survival and optimal surgery timing in cervical fracture patients with ankylosing spondylitis: A national multicentre retrospective study
Source: Front Immunol. 2022 Sep 15;13:971947. doi: 10.3389/fimmu.2022.971947 (PMC9521542; doi:10.3389/fimmu.2022.971947)
Supplement: Supplementary file 2 [file DataSheet_2.docx]

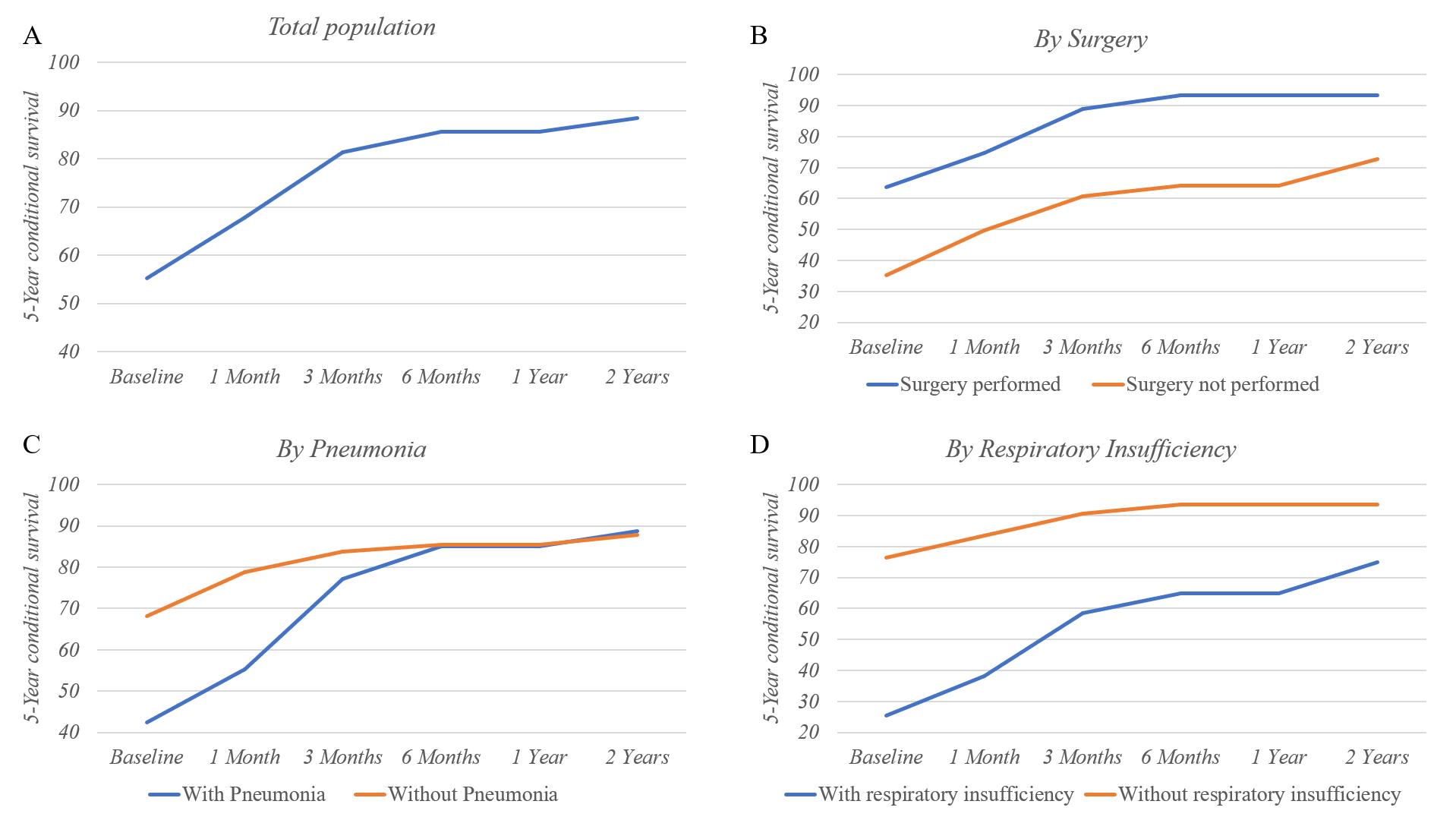


**Supplement Figure 1:** Five-year conditional survival of cervical fracture patients with AS. (A) For ASIA grade A and B populations, (B) stratified by surgery, (C) pneumonia and (D) respiratory insufficiency. AS, ankylosing spondylitis; ASIA, American Spinal Injury Association impairment scale.
